# Microvascular Obstruction in Patients With Anterior STEMI Treated With Supersaturated Oxygen

**DOI:** 10.1016/j.jscai.2024.101356

**Published:** 2024-04-06

**Authors:** Batla Falah, Lak N. Kotinkaduwa, Michael J. Schonning, Björn Redfors, Suzanne de Waha, Christopher B. Granger, Akiko Maehara, Ingo Eitel, Holger Thiele, Gregg W. Stone

**Affiliations:** a Clinical Trials Center, Cardiovascular Research Foundation, New York, New York; b Department of Molecular and Clinical Medicine, Institute of Medicine, Gothenburg University, Gothenburg, Sweden; c Department of Cardiology, Sahlgrenska University Hospital, Gothenburg, Sweden; d Department of Population Health Sciences, Weill Cornell Medicine, New York, New York; e Heart Center Leipzig at Leipzig University and Leipzig Heart Science, Leipzig, Germany; f Department of Medicine, Duke University Medical Center, Durham, North Carolina; g Division of Cardiology, Columbia University Medical Center, New York, New York; h University Heart Centre Luebeck, University Hospital Schleswig-Holstein, Luebeck, Germany; i German Centre for Cardiovascular Research, Luebeck, Germany; j The Zena and Michael A. Wiener Cardiovascular Institute, Icahn School of Medicine at Mount Sinai, New York, New York

**Keywords:** cardiac magnetic resonance imaging, microvascular obstruction, pooled analysis ST-elevation myocardial infarction, supersaturated oxygen therapy

## Abstract

**Background:**

Supersaturated oxygen (SSO_2_) delivered into the left anterior descending coronary artery after percutaneous coronary intervention (PCI) for anterior ST-segment elevation myocardial infarction (STEMI) has been shown to reduce infarct size, but its effects on microvascular obstruction (MVO) are unknown. The aim of this study was to compare MVO in patients with anterior STEMI treated with SSO_2_ after successful primary PCI from 2 studies (the optimized SSO_2_ pilot and IC-HOT) with similar patients from 7 randomized trials who underwent primary PCI without SSO_2_ treatment.

**Methods:**

A total of 874 patients with anterior STEMI who underwent MVO assessment using cardiac magnetic resonance imaging within 10 days after primary PCI were included, of whom 90 patients (10.3%) were treated with SSO_2_. The primary end point was the extent of MVO as a continuous measure in a weighted multivariable model. The secondary end point was the presence of MVO.

**Results:**

SSO_2_ therapy was independently associated with a lower extent of MVO compared with no SSO_2_ therapy (coefficient, −1.35; 95% CI, −2.58 to −0.11; *P* = .03). SSO_2_ therapy was also associated with a borderline lower risk of any MVO (adjusted odds ratio, 0.56; 95% CI, 0.31-1.00; *P* = .051).

**Conclusions:**

In the present individual patient data pooled analysis from 9 studies, SSO_2_ therapy was associated with less MVO after successful primary PCI for anterior STEMI.

## Introduction

Although early reperfusion by primary percutaneous coronary intervention (PCI) has improved the prognosis of patients with ST-segment elevation myocardial infarction (STEMI),^1^, ^2^, ^3^, ^4^, ^5^, ^6^ approximately 20% of patients with STEMI still develop heart failure (HF).^7^ Infarct size and microvascular obstruction (MVO) are both strong predictors of HF and death after STEMI.^8^^,^^9^

The intracoronary infusion of supersaturated oxygen (SSO_2_) in the left anterior descending (LAD) coronary artery after successful primary PCI for anterior STEMI significantly reduced infarct size in the randomized Acute Myocardial Infarction With Hyperoxemic Therapy II (AMIHOT II) trial (NCT00175058) but was associated with a numerical increase in-stent thrombosis events, possibly related to the 90-minute dwell time of the infusion catheter in the LAD.^10^ The delivery system was modified so that SSO_2_ was delivered to the origin of the left main coronary artery for 60 minutes via a diagnostic catheter after successful primary PCI (optimized SSO_2_ delivery). Following the optimized SSO_2_ pilot study,^11^ the safety of optimized SSO_2_ delivery was demonstrated in the prospective single-arm Evaluation of Intracoronary Hyperoxemic Oxygen Therapy in Anterior Acute Myocardial Infarction Patients (IC-HOT) study (NCT02603835), leading to U.S. Food and Drug Administration approval of SSO_2_ therapy for patients with anterior STEMI undergoing primary PCI within 6 hours of symptom onset. In this study, infarct size was consistent with prior studies in which SSO_2_ was delivered by an intracoronary infusion catheter,^10^^,^^11^ and the 1-year clinical outcomes were improved compared with those in other studies of similar patients in which SSO_2_ was not administered.^12^ However, although SSO_2_ therapy has been shown to reduce endothelial cell swelling and induce capillary vasodilation in experimental animal models,^13^, ^14^, ^15^ it has not been assessed whether MVO after primary PCI in patients with STEMI treated with SSO_2_ is reduced.

We therefore sought to examine the presence and extent of MVO in patients treated with optimized SSO_2_ therapy compared with those in patients not treated with SSO_2_ after successful primary PCI for anterior STEMI.

## Methods

### Study design, study population, and definitions

The SSO_2_ treatment group consisted of patients from the optimized SSO_2_ and IC-HOT studies.^11^^,^^16^ The study designs of the optimized SSO_2_ pilot study^11^ and the IC-HOT trial (NCT02603835)^16^ have been previously published. In brief, 20 patients with anterior STEMI in the optimized SSO_2_ pilot study and 100 patients with anterior STEMI in the IC-HOT (single arm) study who underwent successful primary PCI (postprocedural Thrombolysis in Myocardial Infarction [TIMI] 2 or 3 flow) of the proximal or mid LAD within 6 hours of symptom onset) and who did not have cardiogenic shock were treated with 60 minutes of SSO_2_ delivered through a 5F diagnostic catheter seated in the origin of the left main coronary artery. Cardiac magnetic resonance (CMR) imaging was performed between 2 and 7 days after PCI to assess infarct size. MVO was also assessed by analyzing late gadolinium enhancement imaging at a core laboratory (Cardiovascular Research Foundation [CRF]) as previously described.^9^ MVO was denoted by the lack of gadolinium enhancement within the hyperenhanced infarct zone and was expressed as a percentage of the total left ventricular (LV) myocardial mass.

MVO results from these 2 studies were compared to the MVO findings similarly determined from patients with anterior STEMI who underwent LAD PCI without SSO_2_ from 7 trials that have been previously described.^17^, ^18^, ^19^, ^20^, ^21^, ^22^, ^23^ To ensure that the comparator population was similar to the SSO_2_ treatment group, patients were included in this analysis if they fulfilled the following criteria: (1) age 80 years or younger; (2) time from symptom onset to device <6 hours; (3) CMR assessment within 10 days with available data on MVO; (4) Killip class <4 (ie, absence of cardiogenic shock) at presentation; and (5) post-PCI TIMI flow grade 2 or 3.

All patients signed informed written consent for each study. Data from these 9 studies (2 SSO_2_ treatment studies, and 7 SSO_2_ untreated control studies) were pooled into a common database at CRF. ZOLL Circulation, Inc, provided funding to CRF for data analysis. The outcomes were interpreted by the authors and the manuscript prepared independent of the study sponsor.

### End points

The primary end point of interest for the present analysis was the extent of MVO assessed by CMR imaging, measured as a continuous variable, read by a core laboratory. The secondary end point was the presence of any MVO.

### Statistical analysis

Categorical variables are presented as percentages and were compared with the χ^2^ test. Continuous variables are presented as mean ± SD and median with IQR and were compared with the Wilcoxon rank-sum test. The association of SSO_2_ and MVO was calculated using linear regression for the end point of the extent of MVO and logistic regression for the end point of the presence of MVO, each adjusted for a prespecified covariate set known to affect MVO, infarct size, and prognosis after anterior STEMI consisting of age, sex, diabetes, hypertension, current smoking, time from symptom onset to device, and baseline TIMI flow grade ≤1 vs ≥2. All statistical analyses were performed with SAS v9.4 (SAS Institute). A 2-sided *P* value of <.05 was considered statistically significant.

## Results

### Baseline characteristics

**Figure 1:**
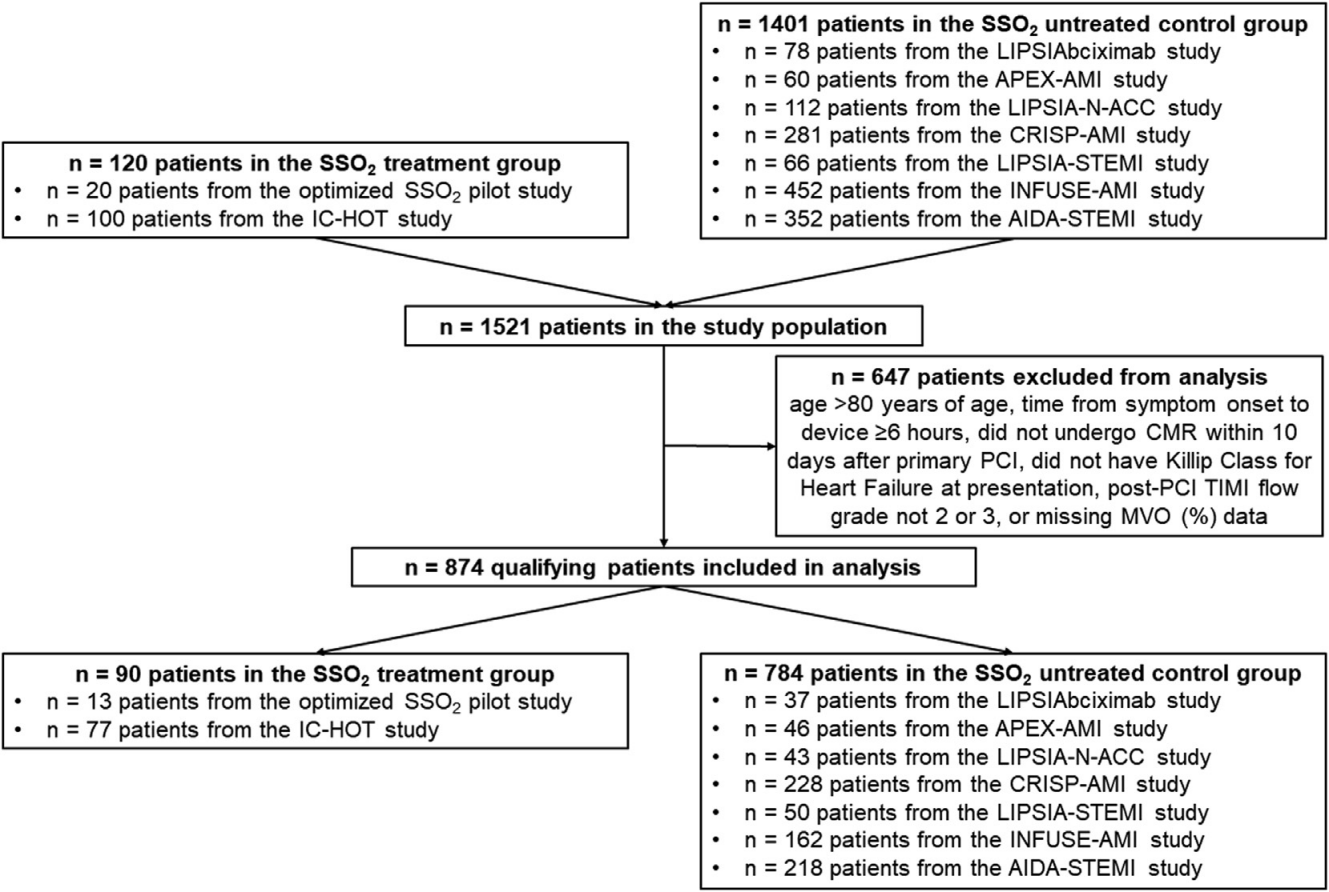
**CONSORT diagram.** CMR, cardiac magnetic resonance; PCI, percutaneous coronary intervention; SSO_2_, supersaturated oxygen; TIMI, Thrombolysis in Myocardial Infarction.

A total of 874 qualifying patients with anterior STEMI who underwent CMR within 10 days after primary PCI and in whom data on MVO were available were included; 90 received SSO_2_ and 784 did not (Figure 1). Features of the individual studies are shown in Supplemental Tables S1 and S2. As shown in Table 1, patients in the SSO_2_-treated group were more likely to be smokers and have diabetes and had shorter duration from the onset of chest pain to device time compared with the control group. There were no differences between the groups in sex, age, hypertension, baseline TIMI flow grade, or final TIMI flow grade.

**Table 1:** Baseline clinical, angiographic, and procedural characteristics in patients treated with and without supersaturated oxygen.

Characteristic	SSO_2_-treated group (n = 90)	Control group (n = 784)	Unadjusted *P*
Male sex	76/90 (84.4)	631/784 (80.5)	.37
Age, y	58.0 [50.0, 65.0]	58.8 [50.0, 67.1]	.83
Current smoker	34/58 (58.6)	332/767 (43.3)	.02
Hypertension	47/90 (52.2)	373/783 (47.6)	.41
Diabetes	22/90 (24.4)	120/783 (15.3)	.03
Symptom onset to device time, h	2.6 [1.8, 3.1]	2.8 [2.0, 3.9]	.01
Baseline TIMI flow grade 0/1	53/90 (58.9)	535/778 (68.8)	.058
Final TIMI flow grade 3^a^	88/90 (97.8)	740/784 (94.4)	.17

Data presented as n/N (%) or median [Q1, Q3], where applicable.

TIMI, Thrombolysis in Myocardial Infarction; SSO_2_, supersaturated oxygen.

a The remainder of patients had TIMI flow grade 2.

### Unadjusted MVO results

As shown in Table 2, the median extent of MVO was 0.2% [0.0%-2.1%] of the LV mass in SSO_2_-treated patients (mean, 1.5% ± 2.3%) and median 0.8% [0.0%-3.8%] of the LV mass in the control patients (mean, 2.9% ± 4.9%; *P* = .052). Any MVO was present in 48 of 90 patients (53.3%) treated with SSO_2_ and in 459 of 784 (58.5%) patients not treated with SSO_2_ (*P* = .35). Infarct size was also lower in SSO_2_ treatment arm (Table 2). Further details of the MVO results in the individual studies are presented in Supplemental Tables S3 and S4.

**Table 2:** Microvascular obstruction in patients treated with and without supersaturated oxygen.

Characteristic	SSO_2_-treated group (n = 90)	Control group (n = 784)	Unadjusted *P*
Time to MVO assessment, d	3.8 ± 1.2	3.6 ± 1.5	.23
Extent of MVO, g	0.3 [0.0, 3.4]	1.1 [0.0, 5.2]	.049
Percent MVO, % LV	0.2 [0.0, 2.1]	0.8 [0.0, 3.8]	.052
Any MVO present	48/90 (53.3)	459/784 (58.5)	.35
Infarct size, % LV	20.8 [10.5, 30.4]	24.7 [14.4, 37.3]	.03

Data presented as n/N (%), mean ± SD, or median [Q1, Q3], where applicable.

LV, left ventricular; MVO, microvascular obstruction; SSO_2_, supersaturated oxygen.

### Adjusted MVO results

After multivariable adjustment, SSO_2_ therapy was an independent predictor of the extent of MVO (coefficient, −1.35; 95% CI, −2.58 to −0.11; *P* = .03) (Table 3 and Central Illustration). SSO_2_ was a borderline predictor of freedom from any MVO, although the difference did not reach statistical significance (adjusted odds ratio, 0.56; 95% CI, 0.31-1.00; *P* = .051) (Table 4). Other predictors of the extent and presence of MVO are summarized in Tables 3 and 4. Propensity-adjusted multivariable analysis resulted in comparable findings (Supplemental Tables S5 and S6).

**Table 3:** Independent predictors of the extent of microvascular obstruction.

Covariate	Coefficient (95% CI)	Adjusted *P*
SSO_2_ (vs no SSO_2_)	−1.35 (−2.58 to −0.11)	.03
Age (per 5 y)	−0.16 (−0.31 to 0.00)	.054
Sex (male vs female)	1.10 (0.29-1.92)	.008
Diabetes	1.36 (0.47-2.25)	.003
Hypertension	−0.36 (−1.02 to 0.31)	.29
Current smoking	−0.62 (−1.29 to 0.06)	.07
Time from symptom onset to device (per 1 h)	0.32 (0.06-0.58)	.02
Baseline TIMI flow grade ≤1 (vs ≥2)	2.08 (1.39-2.76)	<.0001

Estimates and 95% CI were estimated using multiple linear models.

SSO_2_, supersaturated oxygen; TIMI, Thrombolysis in Myocardial Infarction.

**Central Illustration:**
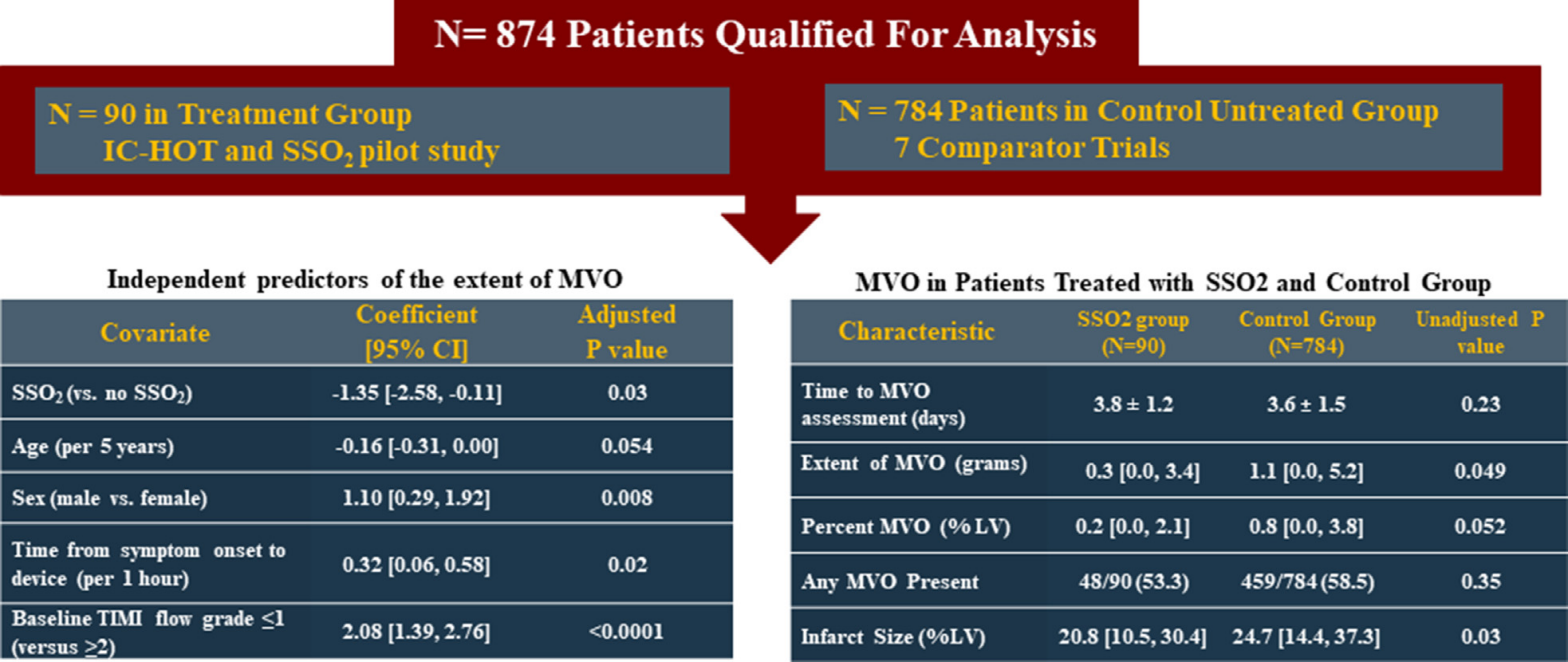
Microvascular obstruction in patients with anterior STEMI treated with supersaturated oxygen pooled analysis. Data are presented as mean ± SD or median [Q1, Q3], where applicable. Estimates and 95% CI are estimated by multiple linear models. LV, left ventricular; MVO, microvascular obstruction; SSO2, supersaturated oxygen; STEMI, ST-elevation myocardial infarction; TIMI, Thrombolysis in Myocardial Infarction.

**Table 4:** Independent predictors of the presence of microvascular obstruction.

Covariate	Adjusted OR (95% CI)	Adjusted *P*
SSO_2_ (vs no SSO_2_)	0.56 (0.31-1.00)	.051
Age (per 5 y)	1.09 (1.01-1.18)	.02
Sex (male vs female)	1.35 (0.92-1.97)	.12
Diabetes	1.59 (1.03-2.45)	.03
Hypertension	0.98 (0.72-1.33)	.88
Current smoking	1.07 (0.78-1.47)	.66
Time from symptom onset to device (per 1 h)	1.03 (0.91-1.16)	.63
Baseline TIMI flow grade ≤1 (vs ≥2)	3.45 (2.51-4.74)	<.0001

Odds ratios and 95% CI were estimated using multiple logistic models.

OR, odds ratio; SSO_2_, supersaturated oxygen; TIMI, Thrombolysis in Myocardial Infarction.

## Discussion

To our knowledge, this is the first study to examine whether post-PCI SSO_2_ infusion, a therapy known to reduce infarct size after anterior STEMI as shown in the AMIHOT II trial,^10^ is also associated with reduced MVO. From this nonrandomized multivariable-adjusted comparison of the outcomes of SSO_2_ therapy in 2 studies vs control patients from 7 studies in which all patients had anterior STEMI treated with primary PCI with MVO assessed by late gadolinium enhancement of an early CMR scan postreperfusion, SSO_2_ therapy was associated with a significant reduction in the extent of MVO and a borderline reduction in the presence of any MVO. These findings likely contribute to the benefit of SSO_2_ therapy in reducing infarct size.^9^

Several pharmacologic and mechanical strategies have been utilized to improve microcirculatory function in patients with STEMI to prevent and reduce the extent of MVO, including mechanical circulatory support, intracoronary adenosine, nitroprusside, and intracoronary abciximab.^17^, ^18^, ^19^, ^20^, ^21^, ^22^, ^23^, ^24^ However, none of these treatments, except possibly for intracoronary abciximab, have been shown to reduce MVO or improve clinical outcomes. The randomized AMIHOT II trial established the ability of SSO_2_ to reduce infarct size. However, technitium-99m sestamibi single-photon emission computed tomography and not CMR was used to assess the degree of myonecrosis in that study,^10^ precluding assessment of MVO. In the nonrandomized IC-HOT study, patients with anterior STEMI treated with SSO_2_ had a low incidence of MVO after primary PCI as assessed with CMR, although the absence of a comparator group challenged the interpretation of this finding.^16^ This study demonstrates that the extent of MVO may be reduced with SSO_2_ after successful primary PCI of the LAD compared with a matched control population, contributing to the reduction in infarct size with this therapy.^9^ Moreover, patients treated with SSO_2_ have been shown to have lower rates of mortality and HF hospitalization at 1-year follow-up compared with a matched control population from the INFUSE-AMI trial treated without SSO_2_.^12^ These data are consistent with those in prior studies that have shown a strong association among MVO, infarct size, and all-cause mortality and HF hospitalization within 1 year.^9^^,^^25^ Thus, this study extends these prior findings; the lesser extent of MVO with SSO_2_ therapy after primary PCI in anterior STEMI likely underlies its mechanism in decreasing infarct size, potentially improving the prognosis of high-risk patients with anterior STEMI.

MVO is associated with infarct size,^9^ a major determinant of freedom from death and HF hospitalization after STEMI.^25^ However, MVO may also adversely affect prognosis through mechanisms that are independent of infarct size.^9^^,^^26^ Specifically, anterior STEMI patients with large extent of MVO (defined as above the median value of 0.47%) were 3.2 times (hazard ratio, 3.21; 95% CI, 1.60-6.46) more likely to die or have a HF hospitalization within 1 year compared patients with small extent of MVO.^9^ The pathophysiology behind these adverse events is multifactorial where MVO limits the delivery of endogenous factors involved in postinfarction remodeling and clearing of cellular debris.^27^^,^^28^ MVO has been associated with increased myocardial stiffness and reduced elasticity, impaired remodeling predisposing the heart to HF and lethal ventricular arrhythmias, as well as with more severe myocardial wall thinning over the first several months after myocardial infarction.^26^^,^^29^^,^^30^ In patients treated with SSO_2_ therapy, the observed extent of MVO of 0.2% is low compared with 0.8% seen in the control group in which SSO_2_ was not administered. Hence, a treatment such as SSO_2_ that appears to reduce MVO may favorably affect prognosis and clinical long-term outcomes through multiple pathways.

### Limitations

This study comprised a nonrandomized comparison of patients from different trials and should be considered hypothesis generating. Despite multivariable adjustments for factors known to affect MVO, infarct size, and prognosis after STEMI, we cannot exclude the presence of unmeasured confounders. For example, data on specific lesion location (eg, proximal LAD vs. mid LAD) were not available from all studies. The present study was limited to STEMI patients with anterior STEMI who presented without cardiogenic shock in whom primary PCI was successful and who survived until CMR. The findings of this study may thus not apply to patients with nonanterior STEMI, those with cardiogenic shock, or in whom PCI was unsuccessful in restoring at least TIMI 2 flow.

## Conclusions

The principal finding from this nonrandomized individual patient data pooled analysis from 9 studies is that treatment with SSO_2_ after successful primary PCI in anterior STEMI was an independent predictor of less MVO as assessed by CMR. As SSO_2_ has also been demonstrated to reduce infarct size after primary PCI in anterior STEMI, an appropriately powered randomized trial is warranted to definitively determine whether SSO_2_ treatment improves long-term clinical outcomes in patients with anterior STEMI who undergo primary PCI.
