# Supplementary material for: Microvascular Obstruction in Patients With Anterior STEMI Treated With Supersaturated Oxygen
Source: J Soc Cardiovasc Angiogr Interv. 2024 Apr 6;3(5):101356. doi: 10.1016/j.jscai.2024.101356 (PMC11307792; doi:10.1016/j.jscai.2024.101356)
Supplement: Supplemental Tables S1-S6 [file mmc1.docx]

**Supplemental Table S1. Baseline clinical, angiographic, and procedural characteristics in patients treated with supersaturated oxygen**

| **Characteristic** | **Optimized SSO_2_ pilot study^11^**  **(N=13)** | **IC-HOT^12^**  **(N=77)** | **Unadjusted P value** |
| --- | --- | --- | --- |
| Sex, male | 11/13 (84.6) | 65/77 (84.4) | 0.99 |
| Age, years | 55.0 [52.0, 62.0] | 58.0 [50.0, 65.0] | 0.74 |
| Current smoker | 4/13 (30.8) | 30/45 (66.7) | 0.02 |
| Hypertension | 8/13 (61.5) | 39/77 (50.6) | 0.47 |
| Diabetes | 4/13 (30.8) | 18/77 (23.4) | 0.57 |
| Symptom onset to device time, hrs | 3.1 [2.7, 3.5] | 2.4 [1.8, 3.0] | 0.06 |
| Baseline TIMI flow grade 0/1 | 7/13 (53.8) | 46/77 (59.7) | 0.69 |
| Final TIMI flow grade 3^*^ | 13/13 (100) | 75/77 (97.4) | 0.56 |

Data presented as n/N(%) or median [Q1, Q3], where applicable. ^*^The remainder of patients are TIMI flow grade 2. TIMI denotes Thrombolysis In Myocardial Infarction. SSO_2_ denotes supersaturated oxygen.

| **Characteristic** | **LIPSIAbciximab^16^**  **N=37** | **APEX-AMI^17^**  **N=46** | **LIPSIA-N-ACC^18^**  **N=43** | **CRISP-AMI^19^**  **N=228** | **LIPSIA-STEMI^20^**  **N=50** | **INFUSE-AMI^21^**  **N=162** | **AIDA-STEMI^22^**  **N=218** | **Unadjusted P value** |  |
| --- | --- | --- | --- | --- | --- | --- | --- | --- | --- |
| Sex, male | 30/37 (81.1) | 39/46 (84.8) | 26/43 (60.5) | 187/228 (82.0) | 39/50 (78.0) | 134/162 (82.7) | 176/218 (80.7) | 0.050 | |
| Age, years | 57  [53.0, 68.0] | 60  [51.0, 69.0] | 64.5  [54.0, 72.6] | 55.2  [48.1, 63.1] | 60.4  [52.9, 69.4] | 58.0  [50.0, 67.0] | 61.0  [51.0, 70.0] | <0.0001 | |
| Current smoker | 21/37 (56.8) | 17/46 (37.0) | 15/43 (34.9) | 79/228 (34.6) | 24/50 (48.0) | 88/161 (54.7) | 88/202 (43.6) | 0.002 | |
| Hypertension | 22/37 (59.5) | 23/46 (50.0) | 33/43 (76.7) | 64/228 (28.1) | 31/50 (62.0) | 50/162 (30.9) | 150/217 (69.1) | <0.0001 | |
| Diabetes | 7/37 (18.9) | 6/46 (13.0) | 8/43 (18.6) | 41/228 (18.0) | 9/50 (18.0) | 13/162 (8.0) | 36/217 (16.6) | 0.16 | |
| Symptom onset to device time, hrs | 3.1  [1.8, 3.7] | 3.1  [2.5, 3.7] | 3.0  [2.1, 4.4] | 3.2  [2.3, 4.3] | 2.3  [1.8, 3.3] | 2.5  [2.0, 3.5] | 2.6  [1.7, 3.8] | <0.0001 | |
| Baseline TIMI flow grade 0/1 | 26/37 (70.3) | 33/44 (75.0) | 24/39 (61.5) | 174/228 (76.3) | 22/50 (44.0) | 120/162 (74.1) | 136/218 (62.4) | <0.0001 | |
| Final TIMI  flow grade 3^*^ | 33/37 (89.2) | 43/46 (93.5) | 39/43 (90.7) | 223/228 (97.8) | 44/50 (88.0) | 154/162 (95.1) | 204/218 (93.6) | 0.054 | |

**Supplemental Table S2. Baseline clinical, angiographic, and procedural characteristics in patients not treated with supersaturated oxygen in the comparator studies**

Data presented as n/N(%) or median [Q1, Q3], where applicable. ^*^The remainder of patients are TIMI flow grade 2. TIMI denotes Thrombolysis In Myocardial Infarction. SSO_2_ denotes supersaturated oxygen.

**Supplemental Table S3. Microvascular obstruction in supersaturated oxygen treated studies**

| **Characteristic** | **Optimized SSO_2_ pilot study^11^**  **(N=13)** | **IC-HOT^12^**  **(N=77)** | **Unadjusted**  **P value** |  |
| --- | --- | --- | --- | --- |
| Time to MVO assessment (days) | 3.4 ± 0.8 | 3.9 ± 1.3 | 0.17 |  |
| Extent of MVO (grams) | 0.0 [0.0, 0.7] | 0.5 [0.0, 3.5] | 0.24 |  |
| Percent MVO (% LV) | 0.0 [0.0, 0.6] | 0.3 [0.0, 2.4] | 0.21 |  |
| Any MVO present | 5/13 (38.5) | 43/77 (55.8) | 0.25 |  |

Data presented as n/N(%) or as mean ± standard deviation or median [Q1, Q3], where applicable. LV denotes left ventricular; MVO, microvascular obstruction; SSO_2_, supersaturated oxygen.

**Supplemental Table S4. Microvascular obstruction in the comparator studies of patients not treated with supersaturated oxygen**

| **Characteristic** | **LIPSIAbciximab^16^**  **N=37** | **APEX-AMI^17^**  **N=46** | **LIPSIA-N-ACC^18^**  **N=43** | **CRISP-AMI^19^**  **N=228** | **LIPSIA-STEMI^20^**  **N=50** | **INFUSE-AMI^21^**  **N=162** | **AIDA-STEMI^22^**  **N=218** | **Unadjusted P value** |  |
| --- | --- | --- | --- | --- | --- | --- | --- | --- | --- |
| Time to MVO assessment (days) | 2.4 ± 1.4 | 3.6 ± 1.3 | 3.3 ± 1.4 | 3.7 ± 1.1 | 3.1 ± 1.4 | 4.6 ± 1.5 | 3.2 ± 1.5 | <0.0001 |  |
| Extent of MVO (grams) | 1.3 [0.0, 4.5] | 0.0 [0.0, 2.0] | 0.8 [0.2, 3.2] | 2.6 [0.0, 8.7] | 5.9 [0.0, 12.2] | 0.7 [0.0, 3.8] | 0.2 [0.0, 2.6] | <0.0001 |  |
| Percent MVO (% LV) | 0.9 [0.0, 4.1] | 0.0 [0.0, 1.5] | 0.6 [0.2, 2.1] | 2.1 [0.0, 7.2] | 3.4 [0.0, 6.8] | 0.6 [0.0, 2.6] | 0.1 [0.0, 2.1] | <0.0001 |  |
| Any MVO present | 25/37 (67.6) | 21/46 (45.7) | 33/43 (76.7) | 143/228 (62.7) | 36/50 (72%) | 90/162 (55.6) | 110/218 (50.5) | 0.002 |  |

Data presented as n/N(%) or as mean ± standard deviation or median [Q1, Q3], where applicable. LV denotes left ventricular; MVO, microvascular obstruction; SSO_2_, supersaturated oxygen.

**Supplemental Table S5. Independent predictors of microvascular obstruction from a propensity adjusted multivariable linear model**

| **Covariate** | **Coefficient [95% CI]** | **Adjusted**  **P value** |
| --- | --- | --- |
| SSO_2_ (vs. no SSO_2_) | -1.33 [-2.56, -0.09] | 0.04 |
| Age (per 5 years) | -0.17 [-0.35, 0.0009] | 0.05 |
| Sex (male vs. female) | 1.10 [0.29, 1.92] | 0.008 |
| Diabetes | 1.77 [-0.02, 3.55] | 0.052 |
| Hypertension | -0.35 [-1.02, 0.31] | 0.30 |
| Current smoking | -0.39 [-1.49, 0.72] | 0.49 |
| Time from symptom onset to device (per 1 hour) | 0.24 [-0.14, 0.63] | 0.22 |
| Baseline TIMI flow grade ≤1 (versus ≥2) | 1.79 [0.49, 3.08] | 0.007 |

Estimates and 95% CI are estimated by Propensity adjusted Multiple Linear models. CI denotes confidence interval; SSO_2_, supersaturated oxygen; TIMI, Thrombolysis In Myocardial Infarction.

**Supplemental Table S6. Predictors of microvascular obstruction from a propensity adjusted multivariable logistic model**

| **Covariate** | **Adjusted OR [95% CI]** | **Adjusted**  **P value** |
| --- | --- | --- |
| SSO_2_ (vs. no SSO_2_) | 0.57 [0.32, 1.02] | 0.057 |
| Age (per 5 years) | 1.08 [1.00, 1.17] | 0.046 |
| Sex (male vs. female) | 1.36 [0.93, 1.99] | 0.19 |
| Diabetes | 1.75 [0.76, 4.04] | 0.19 |
| Hypertension | 0.97 [0.71, 1.32] | 0.85 |
| Current smoking | 1.13 [0.69, 1.86] | 0.63 |
| Time from symptom onset to device (per 1 hour) | 1.01[0.85, 1.2] | 0.90 |
| Baseline TIMI flow grade ≤1 (versus ≥2) | 3.2 [1.81, 5.65] | <0.0001 |

Odds ratios and 95% CI are estimated by multiple logistic models. CI denotes confidence interval; OR, odds ratio; SSO_2_, supersaturated oxygen; TIMI; Thrombolysis In Myocardial Infarction.
